# Near-Complete Genome Sequence of Infectious Bronchitis Virus Strain VFAR-047 (GI-16 Lineage), Isolated in Peru

**DOI:** 10.1128/MRA.01555-18

**Published:** 2019-01-31

**Authors:** Luis Tataje-Lavanda, Ray Izquierdo-Lara, Phillip Ormeño-Vásquez, Katherine Huamán-Gutiérrez, Mirko Zimic-Peralta, Manolo Fernández-Díaz

**Affiliations:** a Laboratorios de Investigación y Desarrollo, FARVET, Chincha Alta, Ica, Peru; KU Leuven

## Abstract

Here, we report the near-complete genome sequence of the infectious bronchitis virus (IBV) strain VFAR-047, isolated in Peru in 2014. This strain was classified into GI lineage 16 (GI-16) based on both the genome and Spike 1 (S1) sequence analysis. Furthermore, four potential recombination events with other GI-16 and GI-11 strains were identified.

## Abstract (precis)

Here, we report the near-complete genome sequence of the infectious bronchitis virus (IBV) strain VFAR-047, isolated in Peru in 2014. This strain was classified into GI lineage 16 (GI-16) based on both the genome and Spike 1 (S1) sequence analysis.

## ANNOUNCEMENT

Infectious bronchitis virus (IBV) (Coronaviridae, Gammacoronavirus) is an economically significant pathogen of the poultry industry worldwide. It causes low egg and meat production and the highly contagious respiratory disease avian infectious bronchitis (1–3) in poultry. IBV has a high mutation and recombination rate, leading to the frequent appearance of new genotypes and antigenic variants worldwide with little or no cross-protection, mainly due to Spike 1 (S1) protein variability (3–5). IBV is classified into six main genotypes (GI to GVI) comprising 32 viral lineages (1 to 32) based on complete nucleotide sequences of the S1 and typical geographical distribution (6). The GI lineage 16 (GI-16), also called the Asia/South America II (A/SAII) genotype, was previously known as the Q1 or CK/CH/LDL/97I type (6, 7). This genotype has been reported in South America (Chile, Colombia, Uruguay, Argentina, and Peru), Asia, and Europe (6, 8–11).

The IBV isolate designated VFAR-047 was isolated from a broiler farm in the north of Lima (Peru) in 2014 (11). Fresh tracheas and kidneys were homogenized and inoculated into the allantoic cavities of specific-pathogen-free embryonated eggs. The allantoic fluid containing IBV was clarified and concentrated in a 20% sucrose gradient by ultracentrifugation. The viral RNA was isolated with an RNeasy midi kit (Qiagen, Germany) and precipitated in ethanol. It was sequenced by Macrogen, Inc. (South Korea) with the HiSeq 2000 platform (Illumina) using the TruSeq stranded total RNA low-throughput (LT) sample prep kit (101 paired ends) (Illumina). The 47,399,834 reads were analyzed, quality checked, and *de novo* assembled using VirusTap (12) and NextGENe (13), producing a genome with a 38% GC content and a coverage of 967.07×. Multiple alignments were performed using Multiple Alignment using Fast Fourier Transform (MAFFT) v7.310 (14), and a phylogenetic tree was generated using the neighbor-joining method in Molecular Evolutionary Genetics Analysis X (MEGA X) (15) with 1,000 bootstrap replications. Recombination analyses were performed using SplitsTree4 v4.14.5 (16) and Recombination Detection Program 4 (RDP4) v.4.95 (17).

The VFAR-047 genome is 27,467 nucleotides (nt) long and was annotated using BLAST comparisons with the nonredundant GenBank database, followed by manual curation. The genome has the typical genetic structure of all IBV strains with 13 open reading frames, organized as follows: 5′-1a-1b-S-3a-3b-E-M-4b-4c-5a-5b-N-6b-3′. The cleavage site of the S protein was typical for A/SAII strains and corresponds to the region between nucleotides 21783 and 21794 (amino acid positions 538 to 541 Arg-Thr-Gly-Arg) (9).

**FIG 1:**
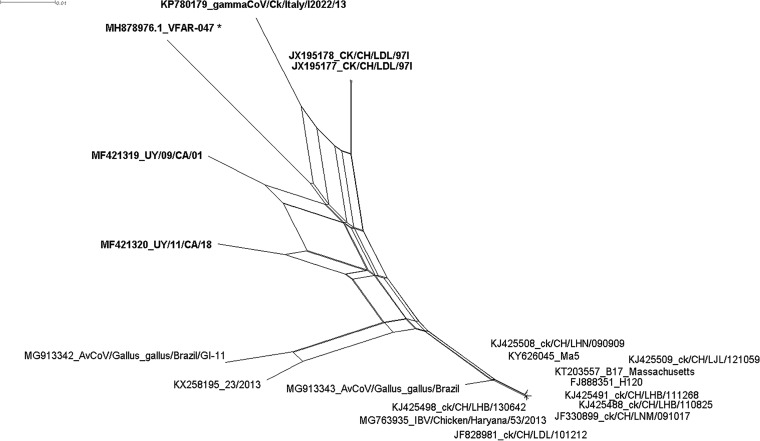
Phylogenetic network (SplitsTree4). The phylogenetic network was constructed using the near-complete genome sequences of 20 IBV strains more closely related to strain VFAR-047 (marked by an asterisk). The multiple reticulate networks indicate historical recombination events (*P* value = 0.0), and the boxes indicate the likelihood of recombination.

The complete S1 gene (1,617 nt) confirmed that VFAR-047 belongs to lineage GI-16, sharing 98% to 93% identity with GI-16 representative strains gammaCoV/Ck/Italy/I2022/13 (GenBank accession number KP780179), ck/CH/LDL/97I (JX195177 and JX195178), UY/09/CA/01 (MF421319), and IZO 28/86 (KJ941019). Phylogenetic analysis of the near-complete genome showed that VFAR-047 is grouped in the same branch with UY/09/CA/01, UY/11/CA/18 (MF421320), gammaCoV/Ck/Italy/I2022/13, and CK/CH/LDL/97I, with average nucleotide identities ranging from 93% to 92%. The phylogenetic network (Fig. 1) and the phi test showed statistically significant evidence of recombination (*P *=* *0.0). Furthermore, five recombined sequences were identified with high reliability by six methods embedded in RDP4, which include four potential recombination events with GI-16 and GI-11 strains.

The VFAR-047 genome will facilitate future development of new vaccines and other infection control strategies in the poultry industry.

### Data availability.

The near-complete genome sequence of infectious bronchitis virus isolate VFAR-047 has been deposited in GenBank under the accession number MH878976. Raw data were deposited in the SRA under BioSample number SAMN10521748 and SRA run number SRR8281084, which are part of SRA study number SRP172861.

## References

[1] World Organization for Animal Health. 2018 Chapter 2.3.2. Avian infectious bronchitis *In* OIE terrestrial manual 2018. World Organization for Animal Health, Paris, France http://www.oie.int/fileadmin/Home/eng/Health_standards/tahm/2.03.02_AIB.pdf.

[2] Cavanagh D 2007 Coronavirus avian infectious bronchitis virus. Vet Res 38:281–297. doi:10.1051/vetres:2006055. 17296157

[3] Lin S-Y, Chen H-W 2017 Infectious bronchitis virus variants: molecular analysis and pathogenicity investigation. IJMS 18:2030. doi:10.3390/ijms18102030. PMC5666712 28937583

[4] Mo M-L, Li M, Huang B-C, Fan W-S, Wei P, Wei T-C, Cheng Q-Y, Wei Z-J, Lang Y-H 2013 Molecular characterization of major structural protein genes of avian coronavirus infectious bronchitis virus isolates in southern China. Viruses 5:3007–3020. doi:10.3390/v5123007. 24304696 PMC3967158

[5] Thor SW, Hilt DA, Kissinger JC, Paterson AH, Jackwood MW 2011 Recombination in avian gamma-coronavirus infectious bronchitis virus. Viruses 3:1777–1799. doi:10.3390/v3091777. 21994806 PMC3187689

[6] Valastro V, Holmes EC, Britton P, Fusaro A, Jackwood MW, Cattoli G, Monne I 2016 S1 gene-based phylogeny of infectious bronchitis virus: an attempt to harmonize virus classification. Infect Genet Evol 39:349–364. doi:10.1016/j.meegid.2016.02.015. 26883378 PMC7172980

[7] Han Z, Sun C, Yan B, Zhang X, Wang Y, Li C, Zhang Q, Ma Y, Shao Y, Liu Q, Kong X, Liu S 2011 A 15-year analysis of molecular epidemiology of avian infectious bronchitis coronavirus in China. Infect Genet Evol 11:190–200. doi:10.1016/j.meegid.2010.09.002. 20833270 PMC7106237

[8] Jackwood MW 2012 Review of infectious bronchitis virus around the world. Avian Dis 56:634–641. doi:10.1637/10227-043012-Review.1. 23397833

[9] Marandino A, Pereda A, Tomás G, Hernández M, Iraola G, Craig MI, Hernández D, Banda A, Villegas P, Panzera Y, Pérez R 2015 Phylodynamic analysis of avian infectious bronchitis virus in South America. J Gen Virol 96:1340–1346. doi:10.1099/vir.0.000077. 25667323 PMC7081071

[10] Marandino A, Tomás G, Panzera Y, Greif G, Parodi-Talice A, Hernández M, Techera C, Hernández D, Pérez R 2017 Whole-genome characterization of Uruguayan strains of avian infectious bronchitis virus reveals extensive recombination between the two major South American lineages. Infect Genet Evol 54:245–250. doi:10.1016/j.meegid.2017.07.009. 28705717 PMC7106025

[11] Tataje-Lavanda L, Falconi-Agapito F, Montalván Á, Bueno C, Requena D, Fernández-Díaz M 2016 First evidence of detection of Asia/South America II (A/SAII) infectious bronchitis virus in a commercial broiler flock in Peru. Vet Rec Case Rep 4:e000292. doi:10.1136/vetreccr-2016-000292.

[12] Yamashita A, Sekizuka T, Kuroda M 2016 VirusTAP: viral genome-targeted assembly pipeline. Front Microbiol 7:32. doi:10.3389/fmicb.2016.00032. 26870004 PMC4735447

[13] SoftGenetics. 2016 NextGEne 2.4.2-Next Generation Sequencing Software for Biologists—User Manual. SoftGenetics, State College, PA.

[14] Yamada KD, Tomii K, Katoh K 2016 Application of the MAFFT sequence alignment program to large data—reexamination of the usefulness of chained guide trees. Bioinformatics 32:3246–3251. doi:10.1093/bioinformatics/btw412. 27378296 PMC5079479

[15] Kumar S, Stecher G, Li M, Knyaz C, Tamura K 2018 MEGA X: Molecular Evolutionary Genetics Analysis across computing platforms. Mol Biol Evol 35:1547–1549. doi:10.1093/molbev/msy096. 29722887 PMC5967553

[16] Huson DH, Bryant D 2006 Application of phylogenetic networks in evolutionary studies. Mol Biol Evol 23:254–267. doi:10.1093/molbev/msj030. 16221896

[17] Martin DP, Murrell B, Golden M, Khoosal A, Muhire B 2015 RDP4: detection and analysis of recombination patterns in virus genomes. Virus Evol 1:vev003. doi:10.1093/ve/vev003. 27774277 PMC5014473

